# Adoption, orchestration, and deployment of artificial intelligence within the National Health Service—facilitators and barriers: an expert roundtable discussion

**DOI:** 10.1093/bjrai/ubae009

**Published:** 2024-06-05

**Authors:** Gerald Lip, Alex Novak, Mathias Goyen, Katherine Boylan, Amrita Kumar

**Affiliations:** University of Aberdeen, Aberdeen, AB25 2ZD, United Kingdom; North East Scotland Breast Screening Programme, NHS Grampian, Aberdeen, AB25 2XF, United Kingdom; Emergency Medicine Research Oxford (EMROx), Oxford University Hospitals NHS Foundation Trust, Oxford, OX3 9DU, United Kingdom; GE HealthCare, Peter-Müller-Str. 24-26, D-40468, Düsseldorf, Germany; Manchester University NHS Foundation Trust (MFT), Manchester, M13 9WL, United Kingdom; Frimley Health NHS Foundation Trust, Frimley, SL5 7SG, United Kingdom

**Keywords:** artifical intelligence, healthcare, NHS, cancer, radiology, platforms, education, training, adaption, adoption

## Abstract

Clinical care, workflow, and patient outcomes within National Health Service (NHS) radiology services will potentially benefit significantly in coming years with the integration of artificial intelligence (AI) into various specialty areas. This article summarizes an expert roundtable discussion, convened by the British Institute of Radiology, comprised experts from clinical, NHS management and industry backgrounds. Facilitators and barriers to both implementation and integration of AI platforms into daily practice are discussed alongside examples including AI in emergency care and breast screening. Issues addressed include the need to generate more real-world evidence that helps developers and service providers to determine the optimal way for patients to reap the benefits of AI with faster reporting turnaround time, faster diagnoses, and treatment times, for example. To ensure the effective and timely wide-scale implementation, adoption requires a national policy drive and national level coordination but specific, local needs of NHS Trusts and any particular service within Trusts will also need to be met. By embedding AI-capabilities into scanners, ultrasound-devices, X-ray equipment, and Picture archiving and communication system (PACS), local services will benefit, while AI applications can streamline workflows across and between departments and potentially Trusts. AI predictive analytics and systems engineering can be used to better manage patient volume and movement. In the short term, education, training, and confidence in adapting and adopting AI in radiology require dedicated local, national, and governmental effort but the future benefits promise to justify the means.

## Introduction

The substantial growth in innovation and the potential of artificial intelligence (AI) technology to reform radiology continues to build a critical mass, within the United Kingdom and abroad.^1–3^ In fact, the potential benefits bestowed upon clinical care, workflow, and patient outcome are potentially so considerable that with considered planning and implementation, the National Health Service (NHS) might significantly benefit from rapidly adopting, orchestrating, and deploying the technology, at scale, to capitalize on the benefits.^4^

Essentially, AI is the use of digital technology to create systems capable of performing tasks commonly thought to require human intelligence, but due to its ability to analyse and synthesize large volumes of complex data, has the potential to make a significant difference in both effectiveness and efficiency across healthcare for patients, clinicians, and systems management.^1^^,^^4^

AI in healthcare takes different forms, largely depending on end use, for example, operational AI deals with scheduling, demand and capacity forecasting hospital operations,^5^^,^^6^ clinical decision support AI that lends to diagnostic support, auto-segmentation and quantification,^7^ or device specific AI that relates to image quality or protocol selection.^8^

However, innovation by its very nature, is not without issues related to smooth integration with existing systems, technologies and cultures both in terms of compatibility and wider acceptance, including the generation of evidence related to robust health economic analysis, governance, and funding to name a few.^9–11^ These issues have resulted in slower uptake in the NHS, and here we discuss some of these issues and how we might negotiate a way around them.

In addressing both the challenges and potential gains of transforming the NHS into an AI-aligned organization, the NHS has established the NHS Artificial Intelligence Lab (NHS AI Lab).^12^ The NHS AI Lab is a collaboration between healthcare providers, government, academics and industry. Its aim is to facilitate the development, as well as safe and ethical incorporation of AI into the NHS, and draw on this resource to meet both the clinical and administrative challenges of such an expansive organization.^12^

There are already more than 500 Healthcare related AI algorithms approved by the US Food and Drug Administration (FDA), and almost 400 of them are radiology based.^13^ However, it is important to note that even with approved, CE-marked, AI software products there is frequently a lack of peer-reviewed evidence of efficacy and generating high quality data to substantiate clinical impact is vital.^14^ With further expansion of computing power, memory capacity and early adoption by centres, AI technology has clear potential to occupy a significant place among the radiology toolset.^13^ The route ahead is visible but daily clinical and radiological practice is still at the start of this transformative journey, and taking tentative steps to undergo AI-driven reform. There is potential to offer a part-solution to the shortage of medical imaging professionals, and the growing workload, including the legacy of backlogged cases in post-pandemic Britain.^15^ AI also promises to facilitate and augment junior medical staff in reading and confirming imaging, reducing error and the need for close supervision, while it offers some assistance with some of the more monotonous, but necessary, tasks.^16^

In March 2023, recognizing the profound influence that AI promises for the practice of medical imaging and image-guided therapies, the British Institute of Radiology (BIR) convened an expert roundtable discussion panel, comprised experts from clinical, science and industry backgrounds. Together, they discussed both facilitators and barriers to the expeditious and efficient implementation of AI technology within radiology across in the NHS, focusing on its potential for adoption, orchestration, and deployment, and this article summarizes the discussion and offers perspectives for the future.

Real-world examples are cited and illustrate how NHS clinicians have already adopted AI in clinical practice, both locally, and nationally, as well as efforts to partner with industry to accelerate these efforts. The focus is largely upon AI in radiology both *per se*, and essentially at the point where radiology interacts with the wider clinical and patient management pathway.

## AI in emergency medicine—high potential but evidence needed to shift AI mainstream

In the near future, it is widely hoped that emergency medicine may draw benefit from the early adoption of AI technology to facilitate diagnosis, decision-making, outcome prediction, and workflow.^17^ The associated datasets derived from electronic health records are vast in scale and rich in resource as a result of the high volume and turnover of patients, along with the frequent use of clinical measurement and diagnostic technology, including imaging. There is a high degree of variability and clinical uncertainty in presentations to the Emergency Department, and together with the need for time-critical decision-making and intervention, as well as the large and evolving clinical workforce with variable skillsets and a rapid staff turnover, this provides an ideal environment for the application of AI-led technologies which may optimize performance of both clinicians and systems in the delivery of acute healthcare.^17–20^

In particular, there has been a groundswell of interest in emergency medicine-relevant AI-assisted image interpretation algorithms, a number of which are already approved by regulators for clinical use,^13^ and aim to support the accurate interpretation of existing imaging modalities.^21^ Most of the evidence to date is based upon multi-case multi-reader studies, which tend to show an overall improvement in diagnostic performance with the use of AI, often driven primarily by significant improvements in the performance of more junior readers.

This phenomenon was demonstrated in a reader study led by the National Consortium of Intelligent Medical Imaging in Oxford which evaluated the impact of the Critical Care Suite (GE HealthCare, Chicago, IL, United States), where the use of an AI-assisted image analysis algorithm was found to significantly improve junior reader proficiency in the identification of pneumothoraces on plain chest X-Ray, yielding comparable diagnostic accuracy to the senior/consultant reader group.^22^ Furthermore, reader performance showed continual improvement with consecutive reads in the second (AI-assisted) phase of the reader study, suggesting the presence of a learning effect secondary to the use of AI, which effectively served as a tutor, available constantly to both guide and check junior clinicians' decisions, reducing error rates among this particular professional group. A number of other applications have also demonstrated promise in this regard from a similar evidence base, including fracture detection,^23–25^ CT head interpretation,^26–28^ and endotracheal tube placement,^29^ with more studies currently underway.^30^^,^^31^ Prospective studies evaluating the impact of these technologies in clinical practice are beginning to emerge,^32^ and National Institute for Health and Care Excellence (NICE) is scheduled to conduct a preliminary assessment of the evidence base for fracture detection software later this year.^33^

Both a reduction in recall for missed fractures and a smoother workflow have clear operational benefits, but in addition, certain missed fractures can have serious consequences for patients causing long term pain and disability. Other pathologies, including cancers on chest X-ray also have significant negative consequences if missed due to human error, not only for patients, but also for staff who can become distressed when they make an error. It should be noted that not all AI algorithms, however, show improvement in performance of assessing radiographs when compared to the reference standard set by human readers. A recent study that looked at 9 commercially available AI products, 2 for bone age prediction based on hand radiographs and 7 for lung nodule detection on chest radiographs, found only 4 of the AI algorithms for detecting lung nodules on chest radiographs showed improved performance. The remaining 5 algorithms showed no evidence of a difference in performance compared to human readers.^34^

In terms of workflow, a recent AI study based on data from a London teaching hospital showed that large data repositories could be successfully leveraged to predict hospital admissions via the emergency department.^35^ Despite some methodological and design limitations, estimates provided information on how many hospital beds would be needed in the next few hours by analysing live data on patients arriving at the emergency department. The tool drew on data on the average number of beds needed on the same day of the week over the previous 6 weeks, and also accounted for patients yet to arrive at hospital, and as such the algorithm provided much more detailed information than conventional methods. Instead of a single figure prediction for the day overall, the tool generated a probability distribution for how many beds would be needed in 4- and 8-hour windows, potentially enabling a more precise and nuanced service response to fluctuations in demand.^35^

## Data quality, standardization, and evidence of value to healthcare systems needed

There may also be benefits to other specific service-oriented or patient-centred outcomes in the use of AI technology, but before any meaningful comparisons can be made, these need to be identified and standardized across different research teams and programmes. Also, robust health economic evaluations are currently lacking and will be key in demonstrating the technology’s value to healthcare systems, and driving, as well as sustaining, adoption.^15^

On the ground, real-world evidence is likely to make a significant difference to uptake, combined with a national database of AI vendors and the evidence generated by each. Of note, real-world evidence generated prospectively will help identify metrics in the early diagnostic phase of a clinical pathway, for example, faster reporting turnaround time, faster cancer diagnosis and treatment times, as well as evidence around patient outcomes, and the broader experiences of patients and clinicians across the care pathway.

Other challenges relate to the fragmented nature of the NHS, that manifests in a non-uniform implementation landscape. Despite operating under the banner of 1 NHS, healthcare institutions largely operate independently, including in terms of clinical processes and procurement, which tend to be locally determined as a response to national guidelines. This can present a challenge when trying to drive wide-scale adoption of new technologies across the NHS.

Continual workforce turnover, in the emergency department and elsewhere, also challenges implementation of an efficient user-AI interface. Rotation of clinicians in different training stages of their career means many are only active in a certain department for a couple of months, while promotion, retirement, alternative career activity, sickness, maternity leave, further compound this staff turnover. This means any new technology, for example, AI-assisted image interpretation which aims to operate at scale needs to be robust and intuitive for clinicians to minimize training requirements and the potential for error or misuse.

Another important consideration is the need to ensure data quality which currently has potential to be a significant limitation in AI development using NHS clinical data. Paper-based handwritten clinical notes taken during ward rounds are commonplace in the NHS and digitizing all of this information will be essential to the successful implementation of AI in the NHS, as we note in the Scottish Breast Screening Service example mentioned below. In part, this explains why innovation has been more successful in areas with large volumes of high-quality, standardized and digitized datasets, for example, in imaging.

## AI in breast radiology—a perspective on deployment from an early adopter

The Industrial Centre for Artificial Intelligence Research in Digital Diagnostics (iCAIRD) based in Aberdeen and Glasgow, Scotland was an Innovate UK funded consortium with over 50 projects and 40 industry partners.^36^ One of the flagship work projects was in building a Trusted Research Environment (TRE) with Canon Medical Systems housed in NHS Grampian and the University of Aberdeen named as SHAIP—Safe Haven A Platform.

Within SHAIP, NHS Grampian, the University of Aberdeen and Kheiron Medical Technologies worked together to apply the AI algorithm called Mia (Kheiron Medical Technologies, London, United Kingdom), mammography intelligent assessment, to analyse a 4-year, anonymized retrospective test data of over 80 000 sets of mammograms (over 320 000 images).

The research output from this analysis had several significant findings including the differential performance of the AI when there were changes in software affecting the image. The AI performed almost as well as humans, had additional interval cancer detection while workforce modelling showed up to 40% workload reduction with an improvement in results turnaround time.^37^

The success of this project led to a further collaboration with Kheiron Medical Technologies, as part of the NHS Health and Social Care Award, to do a prospective evaluation of Mia, working in a live clinical situation with NHS Grampian called Gemini.^38^

NHS information technology (IT) infrastructure also remains an issue. Part of the reason the prospective project has been successful is due to the move of the Scottish Breast Screening Service to a fully paperless environment. This allows for seamless HL7 (international standards for transfer of clinical and administrative data between software applications used by various healthcare providers) integration, electronic messaging and commands between systems. There are many processes elsewhere in the NHS that still remain paper-based needing additional time-intensive, manual steps. Part of user acceptance and adoption is in removing barriers and making the integration and use of AI as simple as possible.

Determining which AI technology is most suitable to meet the specific, local needs of an NHS Trust and any particular service within that Trust is another outstanding issue that needs to be addressed. There is some guidance available, “A Buyer’s Guide to AI in Health and Care” was issued by NHSX in 2020, which sets out 10 questions that Trusts need to consider in order to make well-informed procurement decisions.^39^ However, robust comparative studies are urgently required to both facilitate and expedite such decisions. Most existing studies are of single AI technologies with few comparative studies between multiple AI’s within the same modality, breast or other. Apart from manufacturer’s claims, no reliable method currently exists by which to determine which AI is best suited for any particular intended use.

## AI to support diagnosis and potentially workflow in stroke detection and chest X-ray

In Glasgow, iCAIRD further evaluated the use of AI in early stroke detection with the use of AI to improve acute stroke workflow,^40^ and chest X-ray (BraveCX; Bering Limited, London, United Kingdom), for which FDA approval was achieved in 2023^41^ by Bering, an iCAIRD consortium partner.

The project investigating AI use in detecting early stroke is developing technology aimed at optimizing clinical workflow in the setting of acute ischaemic stroke for patients being considered for intravenous thrombolysis (or other perfusion therapies).^40^ It is focussed on the specification, research, development, and testing of documentation and software for an Acute Stroke Clinical Cockpit, and is using SHAIP to develop machine learning algorithms aimed at streamlining workflow, identifying key treatment (contra)indications, predicting outcomes, calculating risk/benefit scores, all aimed at supporting decision-making by clinicians.

Another AI technology developed by AI developer Brainomix, is e-Stroke AI imaging technology (e-Stroke Suite; Brainomix, Oxford, United Kingdom) that uses AI to automatically process CT and MRI scans and alert doctors in real-time about those patients who would benefit most from mechanical thrombectomy that can have significant benefits in terms of long-term care and disability.^42^^,^^43^ Fast identification of patients who might benefit from thrombectomy means rapid transfer to a specialist centre for the procedure before irreversible brain injury occurs. A study of the technology was conducted by the Oxford Academic Health Science Network (Oxford AHSN), and evaluated use of e-Stroke in 24 hospitals across England. It was found that the average treatment rate in the e-Stroke hospitals was more than 55% higher than the national average, and that mechanical thrombectomy treatment rates rose to 5.7% at e-Stroke hospitals compared to the national average of 3.6%.^44^

BraveCX uses AI-augmented software for Chest X-Ray (CXR) classification and was trained on over 1 000 000 CXRs acquired across diverse clinical settings using the SHAIP. It serves to expedite workflow and improve the accuracy of diagnosis. In pre-clinical studies, BraveCX detected normal chest X-rays with over 95% accuracy and achieved 97% concordance in radiological sign identification with 3 consultant radiologists.^41^ Deployed as either a cloud-based service, directly on premises, or integrated with third-party systems, BraveCX can interface seamlessly with Radiology Information Systems, and outputs the probability of chest X-rays being normal or abnormal, identifies the likelihood of abnormal radiological signs, and highlights them on an image. As such, BraveCX facilitates clinicians in prioritizing chest X-ray reviews.

## National facilitation of AI adoption and integration across the NHS including data management

Reducing the layers of complexity surrounding adoption of AI across the NHS, as well as optimizing internal structures and overcoming barriers promises to expedite its timely uptake. Barriers to adoption include NHS healthcare informatics systems that are currently suboptimal, set against the backdrop of a digital landscape that shows wide variation between Trusts.^45^^,^^46^

Evidence demonstrating improvement in the staff and patient experience as associated with use of AI in specific settings will provide confidence for NHS providers when considering AI solutions. While a positive NICE recommendation is not required for organizations to adopt a specific medical or diagnostic technology—including AI tools, suppliers might consider the rigorous standard of evidence that would be needed for a positive NICE decision as they consider their evidence generation and collection, which includes cost- and clinical-effectiveness data. However, NICE recognizes the difficulty in evidence generation, particularly for small and medium-sized enterprises (SMEs), and has established an Early Evaluation Assessment process, where promising technologies addressing areas of particular need can receive a provisional recommendation which allows them to gather further Real World Evidence while in use.^47^

Ultimately, wide-scale AI adoption requires a national policy drive and national level coordination whereby the NHS and industry work together on joint deliverables. Currently, AI companies work with early adopter Trusts and larger teaching institutions only. The true value of AI lies in assisting areas with a shortage of radiologists or resources, but unfortunately most Trusts lack the capability of deployment, validation and monitoring of AI tools. As such, national level test platforms would find a role here in helping to validate and monitor appropriate tools on a framework for delivery that can be used nationally, overcoming the limitations of Trusts that lack sufficient resources on a local level.

Implementation plans differ markedly by the organization, in part due to the wide variation that currently pervades the digital landscape across NHS Trusts. As such, a 1 size fits all approach is highly unlikely to meet any single Trust’s needs. Effectively, there are underlying systems-based issues that need resolving before AI can be successfully implemented. The anticipated benefits to patients, as well as to clinical and operational workflow that AI promises, might stimulate the fundamental, underlying digital reform required to enable effective implementation.

Being clear and upfront about the specific needs and capabilities of the organization by which AI will be adopted, including at the integrated care system level will also facilitate a smooth integration as services increasingly work across systems to optimize clinical care.

When considering an AI implementation or evaluation process, it may be beneficial to collect cost effectiveness metrics alongside clinical outcomes. Careful consideration of the costs of installation and integration, the fixed price, the ongoing costs, as well as metrics and findings related to staff training and any alteration in the staff time required for an intervention in comparison with existing practice, should be collated to potentially inform the NICE economic evaluation.

Importantly, the NICE evaluation will also seek to incorporate the views of patients and possibly carers, during the implementation process, in addition to those of staff who may be impacted.

Fundamentally, and across all implementation measures, innovators need to ensure that an NHS organization recognizes, through evidence, the positives associated with AI such that Trusts perceive an incoming technology as a benefit and not a threat or risk. One essential component to a positive adoption by the NHS is ensuring the protection of personal data, such that any need for patient data to leave the NHS firewall is minimized, and ideally, an AI algorithm should only operate within the boundaries of the local NHS Trust server (Cloud or on site).

Envisioning and mapping out the flow of data between local Trust data handlers including clinicians and administrative workers will most likely be a requirement for approval according to a Trust’s Data Protection Impact Assessment and Information Governance protocols. The approval process will be expedited by ensuring that the AI algorithm only has access to the minimum data required and that there is no possibility of the data being used for any other unapproved purposes. If sharing is necessary, then personal patient data should be de-identified.

## Augmenting and supporting clinician roles—increasing work satisfaction

Clinicians often express concern around the threat that AI presents to their role, but proponents of AI point out that the technology should augment not replace the human expert.^48^ Some of the greatest gains with AI lie with its potential to increase work satisfaction and limit burn-out, by relieving clinicians of mundane often administrative, non-radiology demands.

Doctors spend an average of 8.7 hours a week on administrative tasks (16.6% of working hours).^49^ Gradually, AI is now taking over these tasks and enabling radiologists to focus on analysing complex cases, applying their experience and training to ultimately nurture better patient outcomes.

There is also a global shortage of radiologists, and as such, overworked radiologists find that fatigue and burnout is a major challenge to individuals and operational matters.^50–52^ Demand for CT and MRI scans for diagnostic purposes has grown by over 5% each year according to 2022 data from the Royal College of Radiologists.^51^

AI has a role to play here in augmenting radiologist output and increasing workforce efficiency. Primarily, triage and prioritization benefit from AI’s ability to identify scans that have more urgent or suspected abnormal findings within the ever-increasing backlog of scans. NHS trials investigating AI’s use in this respect are ongoing for triaging time-sensitive CT head, mammogram, chest X-ray and CT imaging and are receiving dedicated support from a variety of programmes, including, for example, The AI in Health and Care Award.^53^ AI could also provide the opportunity for medical imaging professionals to develop new skills and expertise, and could also lead to the creation of new roles within the medical imaging professions.^54^

The integration of AI into the workflow needs to be as seamless as possible to the user in order to maximize acceptance and uptake. The Edison AI Orchestrator (GE HealthCare, Chicago, IL, United States), is one such workflow management system which helps to simplify the selection, deployment, and use of multi-vendor AI in both departmental and healthcare enterprise workflows at scale. It is designed to seamlessly integrate AI-based clinical applications into the radiology reading workflows that radiologists are already using and are familiar with.^55^

It is also important to note that AI is not the only possible solution to workforce, workload and burnout issues.^56–58^ Attracting new recruits to all professions within the medical imaging field is vital, as is high quality education and training to ensure staff are efficient and effective.^16^^,^^59^ Increasing support for doctors either with allied professionals or by increasing the number of administrative support roles, would be 1 option to ensure clinicians can dedicate their time to patients and are not overburdened with administrative duties.^60^^,^^61^

## Industry and governmental teams and collaborations around AI technology

Development and integration of AI technology is likely to benefit from constructive NHS- industry partnerships, with the NHS taking a provider-agnostic approach. Real world data evaluation might present one such opportunity for the NHS and industry to work together. The benefits of scale provided by the NHS in validating technology provides a pathway to working with companies towards adoption locally and then regionally. This partnership approach in co-development ensures products are suitable for local use.

Accessing funding streams and support from organizations such as the Small Business Research Initiative—an NHS England & NHS Improvement initiative set up to help the NHS access new technologies, and the Accelerated Access Collaborative might be 1 way of furthering this type of activity.^62^ Alternatively, opportunities may exist for leverage of research and/or innovation projects under existing arrangements such as Managed Equipment Services or other commercial contracts.

Any collaborative project between an NHS Trust and the AI industry, would benefit from building a carefully composed project team from the very start.^63^ An industry-NHS project team might include specialists from informatics, information governance, clinically relevant services—managerial and clinical, as well as relevant people from the innovation or transformation department. Together with representatives from AI developers, such joint working optimizes the balanced consideration of all potential issues across the AI implementation and integration pathway from the earliest stages.

Of particular note, patients should also have representation on the project innovation team to ensure their views and needs are considered in the round, possibly even before incorporating company representation.^64^

A pivotal driver for productive AI adoption is to build trust between patients, providers and AI systems and the technologies they interact with. With this in mind, AI developers need to ensure greater diversity of datasets across ethnicities and geographies because validation data has huge value in driving balanced clinical and operational outcomes.^65^ Avoiding bias from the outset is essential in the development of any algorithm and in the successful deployment of AI technologies, and continual evaluation and mitigation of bias should be part of best practice in the development of AI models and tools.^66^

Co-development opportunities can lever the complementary expertise which SMEs/start-ups, healthcare organizations and Global Corporates can bring to AI development and deployment. A typical use case in these projects might allow a small company (with an AI algorithm) to access data and clinical insight from an NHS organization, and evaluate on a platform technology, such as the Edison AI development/deployment system. The use of a platform or application model provides a potential route to commercialization and market access for small companies for whom this would otherwise be a hurdle while helping to overcome issues around roll out and interoperability with variable NHS systems.

The development of sub-national TREs and Secure Data Environments (SDEs) will provide access to larger bodies of good quality data, while also allowing developers, as well as academic and NHS researchers and innovators access to high quality data and analytical platforms for the training and developing of new AI systems.^67^

The SDE should also mitigate any concerns around intellectual property, and information governance because data will not be exported from a single Trust. Data access can be configured such that only approved researchers and developers will be able to access the data for specified uses. Federated learning also offers a possible alternative option to help mitigate concerns surrounding data protection issues.^68^

In the United Kingdom, NHS England and others are developing frameworks to ensure that AI technology is appropriately governed and fit for use in the health service—for example, the NHS AI lab, and specific programmes to develop appropriate regulatory pathways in partnership with the Medicines and Healthcare products Regulatory Agency. Furthermore, it is important to note that the European Union is in the process of adopting into law the AI Act, a comprehensive framework to regulate and constrain the risk associated with AI.^69^ It proposes to categorize AI applications according to the level of risk they pose to the public and regulate them accordingly. This is the broadest AI legislation proposed anywhere in the world and aims to ensure the trustworthiness of AI applications in the region.

## Platforms, education, and integration of all stakeholders across the AI pathway

AI combined with advanced analytics can be applied at different levels of the health system. On the individual level, AI-capabilities can be embedded into scanners, ultrasound-devices, X-ray equipment, and PACS-systems. In radiology departments or private imaging centres, AI applications can streamline workflows. Finally, AI, predictive analytics and systems engineering can be used to better manage patient volume and movement of patients through entire hospitals or even hospital networks.^70^

Education is also key to adoption by individual radiologists and departments, and the generation and accumulation of evidence is 1 essential component of this, alongside familiarity and confidence.^71^ Along with education, emphasis should also be placed on raising awareness of AI and its applications starting from medical school, through radiology training as well as education courses for all healthcare workers, to make them aware and comfortable about the principles of AI as it can be applied to a clinical pathway, alongside safety and data governance topics.^72^^,^^73^

Regarding departmental applications of AI, technology has been investigated that can optimize patient throughput, which in turn determines staffing levels and capacity by predicting a high “no-show” probability of patients^74^^,^^75^ at a certain date by factoring in time of day, weather forecast, and patient profiles, for example, while limiting “no shows” by sending out SMS reminders. Eliminating “no shows” can limit wastage of resources.

At the enterprise or network level, several healthcare providers have partnered with industry to launch an advanced hospital command centre, with predictive analytics and systems engineering to better manage patient safety, experience, volume, and movement of patients. Around 10 such command centres are up and running in the United States, while the first such command centre in Europe opened in late 2022 in Bradford, United Kingdom.^76^

Seamless implementation and integration of the many layers of both hospital workflow and clinical infrastructure requires identification and surmounting of multiple levels of complexity that could present as barriers. Integration and implementation timeframes vary but initial integration should take around 3 months including data governance and assessment of digital capability.

Further challenges arise around the integration of AI with existing systems and with familiarizing staff with the systems available. The integration of AI is time-consuming, often requiring significant input from already stretched IT and radiology resources. In addition, AI interfaces are often suboptimal, with too many clicks or steps which slow the process down and consequently risk loss of appeal and reduce uptake by potential users.

Given AI is often initially focussed on improving 1 part of the healthcare system, there is a risk that AI-based interventions might consequently lead to an inadvertent bottleneck elsewhere on the clinical and/or administrative pathway if integration is piecemeal and does not consider possible downstream effects. For example, an uptick in lung cancer detection might lead to increased pressure in subsequent treatment provision if the wider workflow and resource remained configured based on prior case detection calculations. Another case in point is found with the application of stroke AI that identifies thrombectomy cases faster in peripheral hospitals, however, if ambulances are unavailable to expedite transfer to specialist surgical units, there is limited outcome advantage of using AI. Only once the algorithm is joined up across the entire clinical pathway, the real-world benefit of AI becomes apparent in terms of better outcomes.

In effect, AI needs to address the entire pathway—in the radiology lifecycle from referral management, vetting, prioritization, image acquisition, evaluation, and subsequent prioritization—and not implement interventions prematurely, which risks deterring potential users from adopting the technology.

## Drive change through AI-related strategy

To facilitate cross-organizational adoption, the panels opinion was that agents or ambassadors of change can help to drive the AI agenda, advocating messages around how and what AI can solve and what advantages AI technology offers over and above alternatives. A key suggestion is to identify the points in the clinical and managerial pathway that present an obstacle to smooth workflow and identify how AI can help to overcome them.

In effect, strategy, rather than technology, drives change and nurtures a real paradigm shift across an organization including clinicians, the IT department, and technical staff.

Across the United Kingdom, policy change needs national-level funding and support, but also the development of specific policy at the devolved group level. National level coordination is also required whereby the NHS and industry work together on joint deliverables. In Estonia, Denmark and Israel, where there is significant political activity around digital health, the healthcare systems are primed for digital health, and digital health tools are already in use. Although these countries have different systems to the United Kingdom’s NHS, there may be certain learning points that can be drawn from each system and integrated into the NHS adoption of AI.^77–79^

## Future directions

There is early evidence showing the potential of AI to augment the work of radiologists or healthcare professionals, for example, in triaging and prioritizing suspected abnormal or cancer cases that could lead to faster diagnosis and management. The panel was confident that with the growth in familiarity and confidence around conducting real-world evaluation work using AI, radiologists will soon become AI-augmented to identify all urgent or abnormal cases as soon as scans get done leading to markedly improved health outcomes, cost savings and system efficiencies. However, significantly more robust evidence across a much wider range of uses is needed to build confidence in what is potentially a paradigmatic shift in approach and practice.

In June 2023, the UK government announced a new £21 million fund to roll out AI across the NHS to help diagnose patients more quickly for conditions such as cancers, strokes and heart conditions.^80^ The Department for Health and Social Care (DHSC) say that NHS Trusts will be able to apply to the AI Diagnostic Fund to accelerate the deployment of the most promising AI imaging and decision support tools to help diagnose patients more quickly for conditions such as cancers, strokes, and heart conditions.^80^ The UK government has also announced plans for the NHS AI Lab to pilot an AI Deployment Platform (AIDP), with the aim of providing a model store for multi-vendor AI medical imaging technologies in radiology workflows.^81^ The hope is it will support the safe, efficient and scalable deployment of AI technologies across the NHS.^81^

Looking ahead, the panel’s opinion was that AI and digital investments should focus on 3 key areas, offering both prescriptive (specific recommendations), and predictive (potential future outcomes). These comprise: AI-driven insights to improve both clinical and operational workflows creating the AI enablers for innovation ecosystems to flourish and democratize the build, deployment, and monitoring of AI solutions; monitoring of real-world AI performance to ensure that AI systems remain safe and effective throughout the whole lifecycle; and importantly the provision of facilitation to accelerate AI adoption by building confidence in the systems and technologies users interact with.

There is also a clear need for more robust evidence across the range of use cases proposed, to reassure stakeholders with respect to efficacy of the technologies and hence drive adoption across the NHS, and identify optimal targets in the clinical pathways to achieve maximum benefit for patients and clinicians.

AI developments are leading to significant changes in the medical landscape and these advances are progressing at a rapid pace. As such, education and training in AI for medical professionals is crucial and must happen quickly. It is important that clinicians understand the opportunities, as well as the challenges and limitations, of AI to ensure they continue to provide the best possible care and outcomes for their patients. Such is the importance of the training of medical imaging professionals, so as they are aware of the benefits and risks of AI’s use, that it should be both mandatory and free-of-charge to all who will benefit.

The final view of the roundtable was that AI has the potential to transform medical practice and in this context with the primary aim of early, accurate, and fast diagnosis of disease. AI applications should become additional tools in the armoury for medical imaging professionals and always with the objective of improving patient outcomes and supporting a stronger relationship between clinicians and their patients by freeing-up clinician time and streamlining the patient pathway. There will be challenges in the adoption of new AI technologies but education and training that focuses on the benefits and limitations of AI, and making AI explainable, will help counter and overcome these challenges. This will mean, as health care providers, we can utilize AI in the best way possible and deliver high quality care to our patients.
